# The bone marrow niche components are adversely affected in sepsis

**DOI:** 10.1186/s43556-020-00010-3

**Published:** 2020-10-20

**Authors:** Fan Yin, Han Qian, Caiwen Duan, Botao Ning

**Affiliations:** 1 Department of Pediatric Intensive Care Unit, Shanghai Children’s Medical Center, Shanghai Jiaotong University School of Medicine, 1678 Dongfang Road, Shanghai, China; 2 Department of Translational Institution, Shanghai Children’s Medical Center, Shanghai Jiaotong University School of Medicine, 1678 Dongfang Road, Shanghai, China

**Keywords:** Bone marrow niche, Sepsis, Lipopolysaccharide (LPS), Mouse model

## Abstract

Multiple organ dysfunction is an important cause of death in patients with sepsis. Currently, few studies have focused on the impact of sepsis on bone marrow (BM), especially on the cell components of BM niche. In this study, we performed mouse sepsis models by intraperitoneal injection of LPS and cecal ligation and puncture (CLP). The changes of niche major components in the mouse BM among vascular structures, mesenchymal stem cells and Treg cells were observed and analyzed. The results showed that pathological changes in BM was earlier and more prominent than in other organs, and various cell components of the BM niche changed significantly, of which vascular endothelial cells increased transiently with vascular remodeling and the regulatory T cells decreased over a long period of time. These results indicated that the components of the BM niche underwent series of adaptive changes in sepsis.

## Introduction

Sepsis is life-threatening organ dysfunction due to a dysregulated host response to infection, and is the leading cause of death in intensive care unit (ICU) [1, 2]. Septic shock frequently cause multi-organ dysfunction [3]. Among these affected organs, it is not clear which was affected initially and most significantly. Clinically, we tend to focus on the conditions of infection, sepsis-induced cardiomyopathy, disseminated intravascular coagulation, and the injury to respiratory system, liver and kidney, while ignored alterations of bone marrow (BM) during sepsis, especially the changes in BM niche.

Previous studies have suggested that key organs such as kidney and gut are vulnerable during sepsis [4, 5], interactions between organs during sepsis play a pivotal role in sepsis pathogenesis [3]. Notably, BM plays an equally important part in sepsis [6], which contains hematopoietic progenitor cells and supporting niche cells. Sepsis-mediated BM suppression often leads to myeloid cell differentiation disorders, and myeloid cell dysfunction is associated with acquired immunodeficiency in sepsis [7]. Niche components of BM do not participate in hematopoiesis, but support hematopoiesis and maintain niche homeostasis. About sepsis, there has been so far much research focused on the regulation of the BM hematopoiesis by the niche component [8, 9], but few dealt with the changes of endothelial cells (ECs), mesenchymal stem cells (MSCs) and immune cells in BM niche.

To illustrate the sepsis induced changes in BM niche, and reveal whether BM is more susceptible to sepsis compared with other organs, we performed sepsis models by lipopolysaccharide (LPS) intraperitoneal injection and cecal ligation and puncture (CLP) to map the changes of niche major components during sepsis.

## Results

### BM is earlier and more prominent in sepsis-mediated multiple organs pathological changes

**Fig. 1:**
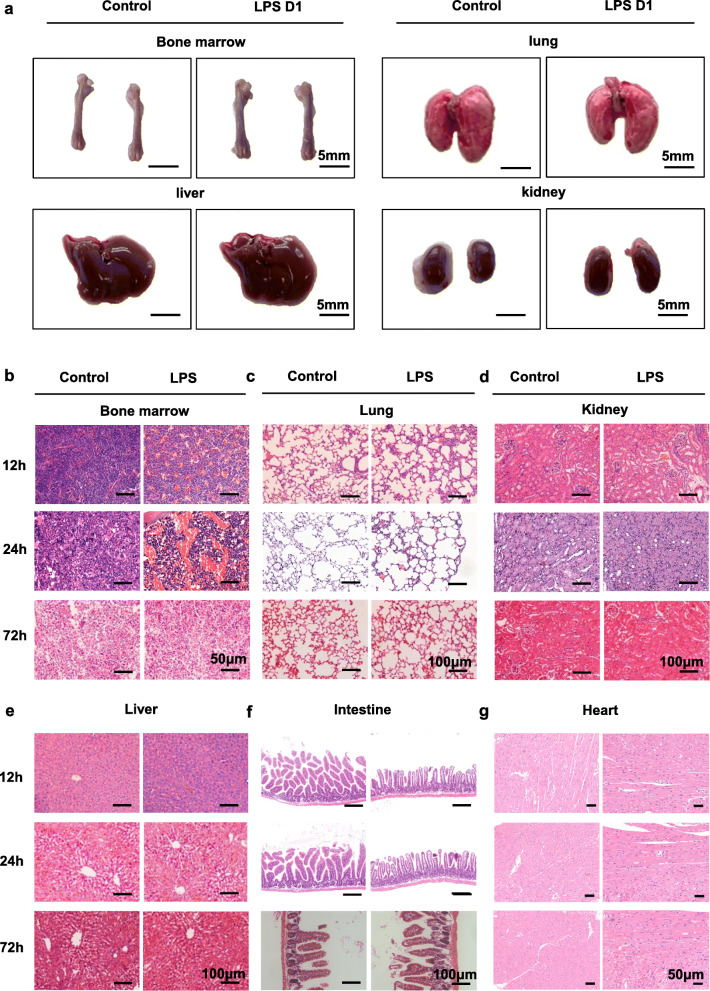
BM undergoes significant histological changes after LPS challenge. **a** Macroscopic images of BM, lung, liver and kidney from 8-week-old control (day 1 after PBS treatment) and LPS D1 (day 1 after LPS treatment) mice. **b, c, d, e, f, g** Histological analysis by H&E staining in the BM (BM) (**b**), lung (**c**), kidney (**d**), liver (**e**), intestine (**f**) and heart (**g**) of mice sepsis model prepared by intraperitoneal injection of LPS at 12 h, 24 h and 72 h after treatment. All representative pictures are verified by independent experiments (n = 3), both control and LPS-treated mice have biological replicates (n>5)

**Fig. 2:**
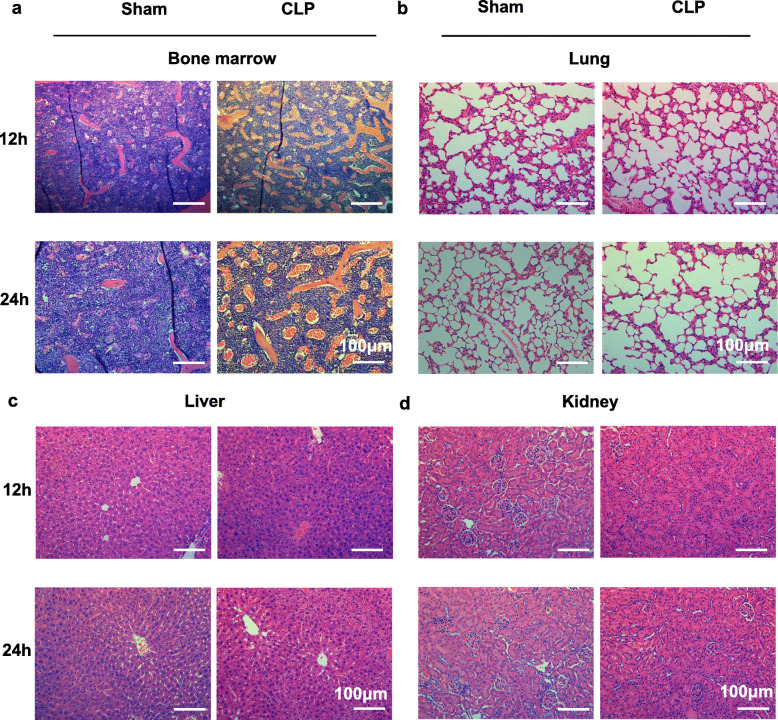
BM in CLP model exhibits similar changes with LPS stimulation. **a, b, c, d** Pictures of H&E staining in the BM (**a**), lung (**b**), liver (**c**) and kidney (**d**) of mice sepsis model prepared by cecal ligation and puncture (CLP) at 12 h and 24 h after operation. All representative pictures are verified by independent experiments (n = 3), both sham-treated and CLP-treated mice have biological replicates (n>5)

To illustrate multiple organ damage in LPS treated mice, we observed the pathological changes of the main organs including BM, lung, kidney, liver, intestine and heart. In LPS treated mice, except for BM congestion, no obvious macroscopic changes were seen in these organs (Fig. 1a). We further studied the pathological changes of major organs in LPS treated mice from the histological level. LPS induced pathological changes in BM mainly occurred in vasculature. Compared with the PBS treated mice, LPS treated mice show obvious hyperemia at 12 h and BM vascular congestion peaked at 24 h and began to subside after 72 h (Fig. 1b). While the lung injury induced by intraperitoneal injection of LPS was relatively mild, and histopathological alterations mainly included pulmonary edema, pulmonary interstitial exudation, neutrophil infiltration (Fig. 1c). Next, we studied the histopathological alterations of the kidney, liver, intestine and heart of mice after LPS challenge and found that there were no obvious pathological changes in these organs (Fig. 1d-g). Taken together, BM was more susceptible to inflammation and exhibited histopathological alterations, which were different from other organs in early sepsis. Concomitantly, the histopathological changes of the main organs in mice after CLP were similar with those after LPS stimulation (Fig. 2a-d).

### Sepsis induces transient BM vasculature remodeling and BM EC proliferation

**Fig. 3:**
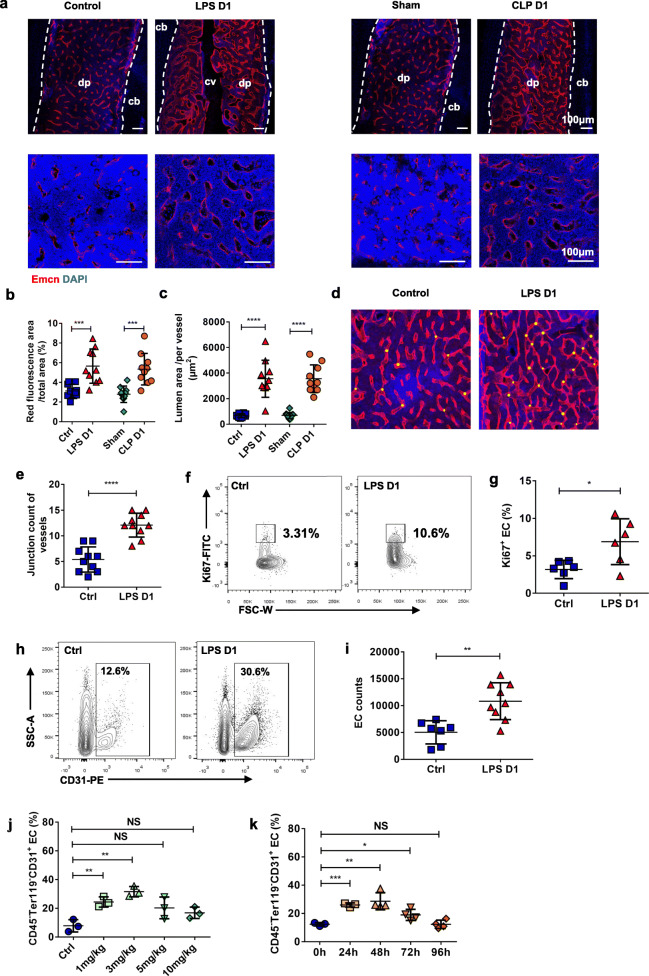
Sepsis alter the BM Vasculature remodeling. **a** Representative images of the femur diaphysis stained with anti-endomucin (Emcn) antibody and 4′,6-diamidino-2-phenylindole (DAPI). Control (day 1 after PBS treatment), LPS D1(day 1 after LPS treatment), Sham (day 1 after sham operation), CLP D1 (day 1 after CLP procedure). Diaphysis (dp), compact bone (cb), central vein (cv). **b, c** Quantification of BM vessels on confocal images of the femur diaphysis showed in (**a**). Ten mice each group, 3 sections per mouse. **d** Visualization of femur vessels junctions (yellow dot) in histological images. **e** Quantitative analysis of junction count of BM vessels showed in (**d**) between control (n = 10) and LPS-treated (n = 10) mice. Three sections per mouse. **f** Ki67 staining for detecting proliferation rates of BM ECs. **g** Percentage of Ki67^+^ BM ECs in controls (n = 6) and treated mice 1 day after LPS injection (n = 6). **h** Representative figures shown the percentage of CD45^−^ Ter119^−^ CD31^+^ ECs in BM. **i** Percentage of CD45^−^ Ter119^−^ CD31^+^ BM ECs in controls (n = 7) and treated mice 1 day after LPS injection (n = 9). **j** Changes of percentage in BM ECs at different concentrations of LPS administration. Three mice in each treatment. Control (Ctrl, PBS treatment). **k** Changes of percentage in BM ECs at different time after 10 mg/kg LPS administration. Three mice in oh and 24 h, 4 mice in 48 h, 72 h, 96 h. All data represent as means±s.d. *p<0.05, **p<0.01, ***p<0.001, ****p<0.0001, as determined by Student’s t-test. NS, not significant

BM vasculature changed drastically in previous histological analysis. To further monitor the structure of the BM vasculature, we specifically labeled the BM vessels by immunostaining. After 24 h, compared with PBS-treated mice, BM vasculature increased significantly in diaphysis regions of LPS and CLP treated mice as intuitively showed in anti-Endomucin staining of frozen BM sections (Fig. 3a). To quantify the LPS and CLP mediated BM vessel remodeling, we evaluated the BM vessel fluorescent area labeled by endomucin and the lumen area of the BM vessel. We found an increased fluorescence area and enlarged vasculature emerged 24 h following related treatment (Fig. 3b, c), a finding that correlated well with an increased branch in BM vasculature patterns of LPS treated mice (Fig. 3d, e). These support the obvious structural remodeling of BM vessels within a short time after LPS and CLP stimulation.

ECs are important cell types involved in vascular remodeling [10]. FACS analysis was used to determine the status of BM vascular ECs after LPS injection. We observed a rise in proliferating Ki67^+^ BM ECs 24 h after LPS injection (Fig. 3f, g), indicating that cells in S-phase increased. Consistent with this, there was a significant increase in frequency and absolute number of BM CD45^−^Ter119^−^CD31^+^ ECs of LPS treated mice in comparison to mice treated with PBS at 24 h (Fig. 3h, i). When mice were given sufficient time to recover after treatment, upregulation of BM ECs returned to homeostatic levels after 96 h (Fig. 3j). This indicated that the response of ECs to LPS treatment is transient. We further found even low dose (1 mg/kg) of LPS exposure could still activate BM ECs (Fig. 3k). This indicates that LPS stimulation activates ECs in a dose-independent manner and that BM ECs were activated even in response to low doses of treatment. Taken together, proliferation of BM ECs is one of the main causes of LPS-mediated BM vascular remodeling.

### Sepsis did not significantly affect the frequency and location of BM MSCs

**Fig. 4:**
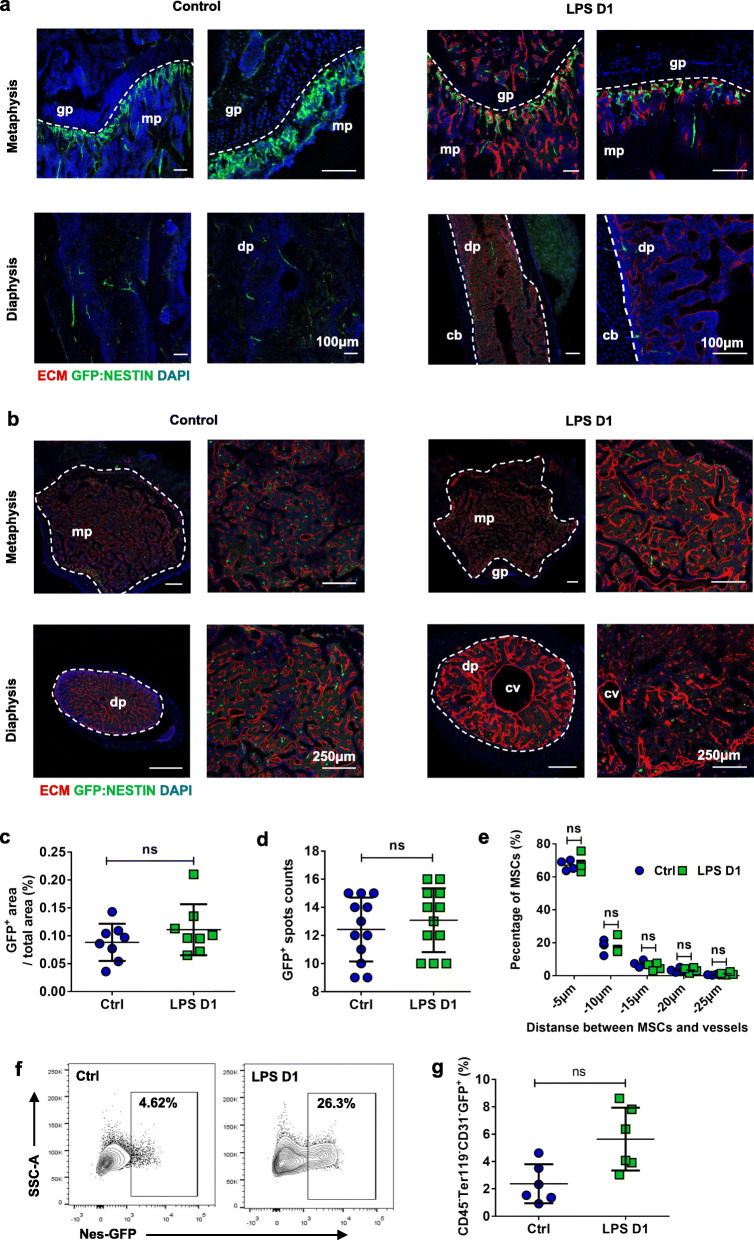
BM MSCs show no significant changes during sepsis. **a**, **b** Longitudinally (**a**) and transverse-shaved (**b**) confocal images of femur metaphysis and diaphysis stained with anti-Emcn antibody and DAPI in control (PBS treatment) and LPS-treated Nestin-GFP transgenic mice. Diaphysis (dp), metaphysis (mp), central vein (cv), growth plate (gp). **c**, **d** Quantification of BM GFP^+^ MSCs on enlarged view showed in (**b**). Eight mice each group, 3 sections per mouse. **e** Quantification of distance between MSCs and BM vessels from femur transverse-shaved sections of control (PBS treatment) and LPS-treated Nestin-GFP mice 1 day after intervention. **f** Representative FACS figures showed the percentage of BM MSCs in control (PBS injection) and LPS-treated Nestin-GFP transgenic mice 1 day after intervention. **g** Percentage of CD45^−^Ter119^−^CD31^−^GFP^+^ MSCs in control (n = 6) and LPS treated (n = 6) mice 1 day after intervention. All data represent as means±s.d. NS, not significant

To illustrate the effects of sepsis on BM perivascular stromal cells enriched in MSCs (MSC) activity, we used mice expressing GFP under the direction of nestin promoter (Nestin-GFP) [11]. Compared with mice treated with PBS, the femur vertical sections in mice treated with LPS revealed that the intensity and density of GFP fluorescence in both the epiphysis and diaphysis regions showed no obvious changes (Fig. 4a), which was consistent with the femur cross section slides in LPS treated mice (Fig. 4b). To quantify the LPS mediated MSCs changes, we evaluated the GFP fluorescent area and MSC spots counts of the BM cross section slides, and we founded there was no significant increase in both GFP fluorescent area and MSC spots counts after LPS treatment (Fig. 4c, d). Meanwhile, we also analyzed the spatial relationship between MSCs and vessels in BM and founded that the spatial distribution of MSCs was not affected by LPS stimulation, which mainly distributed around BM vessels (Fig. 4e). We next analyzed the frequency of MSCs using FACS and observed no significant increase in CD45^−^Ter119^−^CD31^−^GFP^+^ MSCs 24 h after LPS treatment (Fig. 4f, g), which is consistent with the visualization of BM frozen sections. Taken together, BM MSCs did not respond to LPS stimulation at the cellular and tissue level. In addition, the MSCs still mainly distributed around the vessels, close to the BM ECs.

### Sepsis-mediated BM immune changes have a different immunophenotype than the periphery

**Fig. 5:**
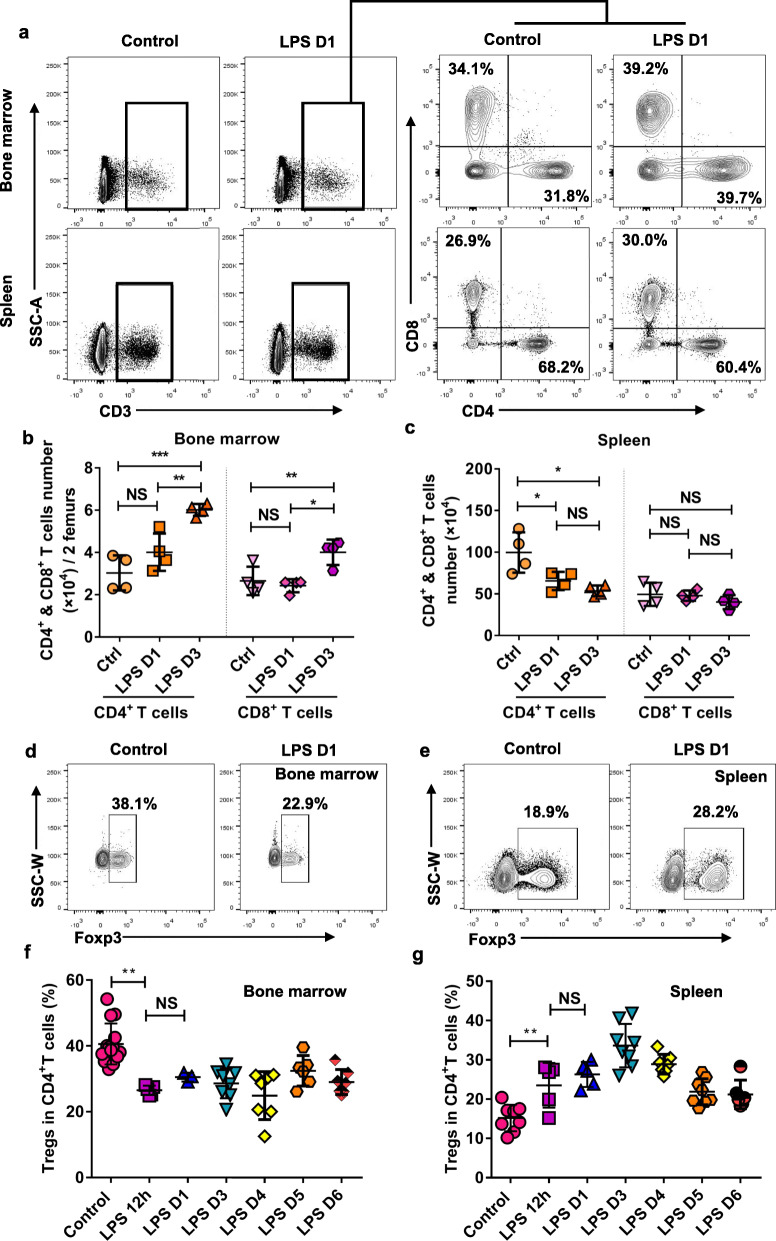
BM immune cells exhibit different characteristics from the periphery and show complexity changes during sepsis. **a** Representative figures shown the percentage of CD4^+^CD8^−^ and CD4^−^CD8^+^ T cells in the BM and spleen of control (PBS-treated, n = 4) and LPS-treated mice (n = 4) at day 1. **b, c** Absolute cell counts of CD4^+^CD8^−^ and CD4^−^CD8^+^ T cells in BM (**b**) and spleen (**c**) as shown in (**a**) at day1 and day 3. **d**, **e** Representative figures shown the percentage of CD3^+^CD4^+^FOXP3^+^ regular T cells (Tregs) in the BM (**d**) and spleen (**e**) of control (PBS-treated) and LPS-treated mice at day 1. **f, g** Dynamic changes of the percentage of Treg cells within a week after LPS treatment. All data represent as means±s.d. *p<0.05, **p<0.01, ***p<0.001, ****p<0.0001, as determined by Student’s t-test. NS, not significant

In our research, we focused on the changes of BM T lymphocytes subsets, especially regulatory T cell. After 24 h, LPS treated mice had a higher level of CD3^+^CD4^+^ T cells and CD3^+^CD8^+^ T cells in BM compared with PBS treated mice, meanwhile the absolute numbers of these two cells also increased significantly in LPS treated mice (Fig. 5a, b). On the contrary, CD3^+^CD4^+^ T cells in spleen of LPS treated mice show a significant increase while CD3^+^CD8^+^ T cells showed no obvious changes. Taken together peripheral lymphocytes subsets reveal a different pattern of immune response from BM (Fig. 5a, c).

We further analyzed the changes of Treg cells, an important inhibitory component of CD4 lymphocytes subsets in spleen and BM after LPS treatment. It is worth noting that BM Treg cells and spleen Treg cells also showed different response patterns after LPS stimulation. The percentage of BM FOXP3^+^ Treg cells decreased significantly in LPS treated mice compared with PBS treated mice (Fig. 5d, f). Inversely, FOXP3^+^ Treg cells showed an increase in spleen of mice treated with LPS (Fig. 5e, g). Notably, unlike the transient changes of BM ECs, the LPS induced Treg cells changes in BM and spleen are more durable. These results collectively suggest that the BM immune niche has a different response pattern from the peripheral immune organ in sepsis.

## Discussion

Sepsis-mediated changes in BM niche components could have a profound effect on hematopoiesis, and may also be one of the pathogenesis of sepsis. In this study. We describe the significant changes of major components in BM niche during early stage of sepsis.

One of the challenges in research related to sepsis is the limitations and variability of existing models of sepsis in simulating human pathophysiologic conditions [12]. CLP is the preferred approach for modeling sepsis, reflecting progression of sepsis characterized with systemic inflammatory response and the compensatory anti-inflammatory response [13]. LPS is the main glycolipid present in the outer leaflet of the outer membrane of Gram-negative bacteria which can induce a strong inflammatory response. We found that organic pathological changes in mice were similar between the two models, and major pro-inflammatory factors up-regulated significantly. In addition, lethal dose of LPS injection usually induces severe inflammatory response and aggravates organ damage, allowing better observation of phenotypes in early stage of sepsis. In this study, sepsis model was mainly performed by lethal does LPS intraperitoneal injection. Because it is worth noting that LPS-induced sepsis model fit the early stage of sepsis and the surviving mice gradually recovered from LPS challenge in the later period (several weeks).

BM ECs are the gateway against infection, so understanding the impact of inflammation on the BM vasculature is essential. Some evidence suggests that the BM vasculature undergoes significant remodeling under LPS stimulation [14]. We found for the first time that after LPS stimulation, BM ECs responded quickly, with a transient increase, and this change did not depend on the dose of LPS administered. The rapid and transient activation of BM ECs may be an emergency response to inflammatory signals from the hematopoietic system, and this effect may in turn promote the maintenance of BM homeostasis. In addition, this transient change in ECs may also have temporal and spatial consistency with certain physiological processes during inflammation. For example, neutrophils migrate from the BM to the periphery during inflammation [15]. Platelet activation and VEGF signaling are the basic mediators of EC activation during inflammation [16, 17]. However, in our study, VEGF expression in ECs was not up-regulated with LPS stimulation. This may indicate that endothelial-derived VEGF is not a major factor in the remodeling of the vasculature during inflammation. This regulation may come from other cells in the BM and platelets.

MSCs are major components of the BM niche, which contain multiple and overlapping populations depending on different markers and genetic reporters [18]. Nestin-GFP as one of the genetic reporters is used to label and trace MSCs [11]. In our study, we used nestin-GFP transgenic mice to assess the effect of sepsis on MSCs in niche. Unlike hematopoietic stem cells, MSCs cannot be mobilized into the peripheral circulation which respond to inflammatory signals generated locally or systemically in situ [19]. MSC senses inflammation stress through pattern recognition receptor (PRR) modifying the cytokine and chemokine profile in BM stroma, which result in a series of adaptive effect including hematopoietic changes [20], mobilization of HSCs [21], monocyte release [9] and inhibition of neutrophil apoptosis [22]. The importance of MSCs as a component of the niche under inflammation condition on stroma remodeling and hematopoietic support is self-evident. However, little is known about the effects of inflammation on MSCs. We report no significant increase in frequency and absolute number with unchanged spatial localization of MSCs during sepsis. MSCs appear to be in a resting state of morphology, which is not consistent with their functional activation during inflammation. In addition, our study failed to address the changes in MSCs throughout sepsis.

Immunosuppression is one of the most important causes of mortality in sepsis [23], which is related to the inappropriate increase of Treg cells and the enhanced function [24, 25]. BM Tregs are an important niche component [26–29] and represent a unique T-cell lineage that the frequency of BM Tregs is significantly increased compared to the periphery [30, 31], and exhibits high inhibitory activity. TIGHT and CD44 and CXCR4 were highly expressed in BM Tregs [30]. BM Treg has been considered to be involved in the pathophysiological processes of various diseases, such as GVHD and prostate cancer [31, 32]. Whether BM Tregs functions in sepsis remain unclear. We observed a significantly decreased frequency of BM Tregs and an increased proportion of CD4^+^CD8^+^ effector T cells in BM during sepsis. Notably, the peripheral T cell subsets changed completely opposite to BM. Tregs are known to be regulated by various chemokine regulatory axes, MSC in BM produce high levels of CXCL12 [30, 31], activated Tregs can migrate through their enhanced CXCR4 expression [33] and eventually reside in the BM. The up-regulated expression of G-CSF in sepsis will reduce the concentration of BM CXCL12, which may cause the unstable of CXCL12/CXCR4 axis [34]. The disruption of this pathway mobilizes Tregs into the periphery, which account for the reduction of BM Tregs and the differentiation and expansion of effector CD4^+^ and CD8^+^ T cells. It should be noted that sepsis-induced BM Treg changes may lead to the destruction of the HSC immune privilege status [35], resulting in a series of hematopoietic problems in the BM under the inflammatory state.

Our findings map the response of major BM niche components to sepsis, which reflects in the earlier and more prominent histological changers than other organs and urgent mobilization of non-hematopoietic cells, such as ECs and Tregs, in the BM under sepsis. We put forward a hypothesis that BM may be the initiating or accelerating organ during sepsis. Future studies will delve into the cellular changes of niche components and study the relationships among BM niche components and peripheral organic microenvironment under the inflammatory state, which may reveal the critical mechanism of septic onset and progress.

## Materials and methods

### Animals

Sepsis models were performed in several mouse strains: adult C57BL/6 mice (Shanghai SLAC Laboratory Animal Co., Ltd., Shanghai, China); Nestin-GFP and Sca1-GFP transgenic mice (The Jackson Laboratory, Bar Harbor, ME, USA). Sex-matched mice of both sexes between the ages of 8 and 14 weeks were used.

### Sepsis model

Sepsis model was performed by LPS intraperitoneal injection [36] and cecal ligation puncture (CLP) according to the methods described in previous studies [8, 13]. Briefly, for CLP model, cecum was ligated at half the distance between distal pole and the base of the cecum with 18 G needle to induce abdominal infection, resulting in a mortality of 60% on the seventh day. The control group underwent exactly the same procedures except for the CLP. For LPS intraperitoneal injection model, mice were subjected to intraperitoneal injection of 10 mg/kg of *E. coli* serotype O111:B4 lipopolysaccharide (LPS) purchased from Sigma (L 4130). Mice in control group was injected with PBS which was used to dissolve LPS. The survival curves of both models were highly matched.

### Hematoxylin-eosin staining

For histology, dissected femurs were fixed in 10% neutral buffered formalin overnight, followed by decalcification in 10% EDTA for 2 weeks. Femurs were embedded in and then sectioned at 6 μm thickness. Hematoxylin and eosin (H&E) staining was performed according to a standard procedure [37].

### Immunostaining

Immunostaining was performed according to a published procedure [38]. Briefly, dissected femurs were fixed with 4% paraformaldehyde (PFA) at 4°C overnight and decalcified in 0.5 M EDTA for 24 h with constantly shaking. The femurs were dehydrated with 20% sucrose at 4°C for 24 h then embedded and stored at − 80°C overnight before section. Femurs were sectioned horizontally and longitudinally at 40 μm thickness using Leica cryostat (Leica, CM1950). For immunostaining, femur sections were stained with Endomucin (anti-rat, sc-65,495, Santa Cruz Biotech.). The primary antibodies were bound by secondary antibodies (AF555 goat anti-rat, 2,018,295, Life technologies). Nuclei were stained with DAPI (62,248, Life Technologies). Images were acquired with a confocal microscope (Leica SP8) and analyzed with ImageJ and Angio Tool software.

### Flow cytometry

For flow cytometry of ECs and MSCs, bones (Tibia, femur, sacrum) were crushed and digested with 1 mg/mL collagenase IV and 2 mg/mL dispase in phosphate buffer saline (PBS) containing 1% BSA at 37°C for 1 h. For immune cells, bones were flushed with pre-chilled PBS. For Single-cell suspensions of peripheral immune organs, lymph nodes and spleens were crushed gently on filter. Collected cells were incubated with Fc blocker anti-CD16/32 antibody (93, 14–0161-82) for 15 min on ice, followed by staining with fluorochrome-conjugated antibody on ice for 45 min. The antibody used in this study included FITC/APC anti-CD45 (30-F11, 11–0451-85/17–0451-82); FITC/APC anti-Ter119 (Ter-119, 11–5921-82/17–5921-82); APC/PE anti-CD31 (390, 12–0311-82/17–0311-82) (all from ebioscience); DAPI (4′,6-diamino-2-phenylindole) (62,248, Life Technologies) was used to exclude dead cells. BD FACS Canto II flow cytometer equipped with the FACS Diva 6.1 software (BD Biosciences) were used to collect samples information. Data were analyzed with FlowJo version 10.

### Statistical analysis

All data are measurement data and represented as mean ± s.e.m. Normality analysis (Kolmogorov–Smirnov tests) and homogeneity test of variance (Levene tests) were done between different samples, Comparisons between two samples were done using the unpaired Student’s t tests. One-way ANOVA analyses followed by Student-Newman-Keuls multiple comparison tests were used for multiple group comparisons. Wilcoxon tests and Kruskal-Wallis tests were used for samples with inconsistent distributions and variances. Statistical analyses were performed with GraphPad Prism 6 and Statistical Product and Service Solutions (SPSS) version 20 software. *p < 0.05, **p < 0.01, ***p < 0.001.

## Data Availability

All data generated or analyzed during the current study are available from the corresponding author on reasonable request.

## Abbreviations

FACS: Fluorescence activated cell sorting
BM: Bone marrow
LPS: Lipopolysaccharide
CLP: Cecal ligation and puncture
ECs: Endothelial cells
GFP: Green fluorescent protein
MSCs: Mesenchymal stem cells
Tregs: Regulatory T cells
FOXP3: Forkhead box protein 3
