# Author Correction: The bone marrow niche components are adversely affected in sepsis

**DOI:** 10.1186/s43556-025-00304-4

**Published:** 2025-11-04

**Authors:** Fan Yin, Han Qian, Caiwen Duan, Botao Ning

**Affiliations:** 1 Department of Pediatric Intensive Care Unit, Shanghai Children’s Medical Center, Shanghai Jiaotong University School of Medicine, 1678 Dongfang Road, Shanghai, China; 2 Department of Translational Institution, Shanghai Children’s Medical Center, Shanghai Jiaotong University School of Medicine, 1678 Dongfang Road, Shanghai, China

**Correction** **:** **Mol Biomed 1, 10 (2020)**

**https://doi.org/10.1186/s43556-020-00010-3**

Following publication of the original article [1], it is reported that there were errors in Fig. 1d, 1g, 2a, 3a, 4b and 4f, which resulted from an inadvertent discrepancy between source images and figure labels that occurred during file preparation, leading to non-corresponding visual data in the published version.

In Fig. 1d, the two images in 72h kidney control and 72h kidney LPS were incorrect and from another experimental replicate of 24h kidney control. These two images require correction. In Fig. 1g, 72h heart LPS image was incorrectly formed from 24h heart LPS image, and thus needs to be corrected.

In Fig. 2a, 24h Sham image was incorrect and belonged to 12h Sham image. Image in 24h Sham should be revised for accuracy.

In Fig. 3a, the control (up) and CLP D1 (down) panels were duplicates of the correct Sham (up) and LPS D1 (down) images, respectively, resulted from a layout preparation error. Images in control (up) and CLP D1 (down) require replacement. The quantitative data in Fig. 3b and Fig. 3c (histograms) remain valid and correctly represent the experimental findings.

In Fig. 4b, the control diaphysis (right) is histologically confirmed to be the metaphysis, while the control metaphysis (right) actually shows the diaphysis. Modification is required to correct the image-label correspondence. These misassigned image-label pairs are limited to control groups, thus the statistical outcomes in Fig. 4c and Fig. 4d remain unaffected. In Fig. 4f, the representative flow cytometry plot for LPS D1 was inadvertently selected from an outlier dataset and requires replacement to reflect the predominant population distribution. The original and corrected figures are shown below. These corrections do not affect the findings or conclusions of this study.

Incorrect Fig. 1:

**Fig. 1:**
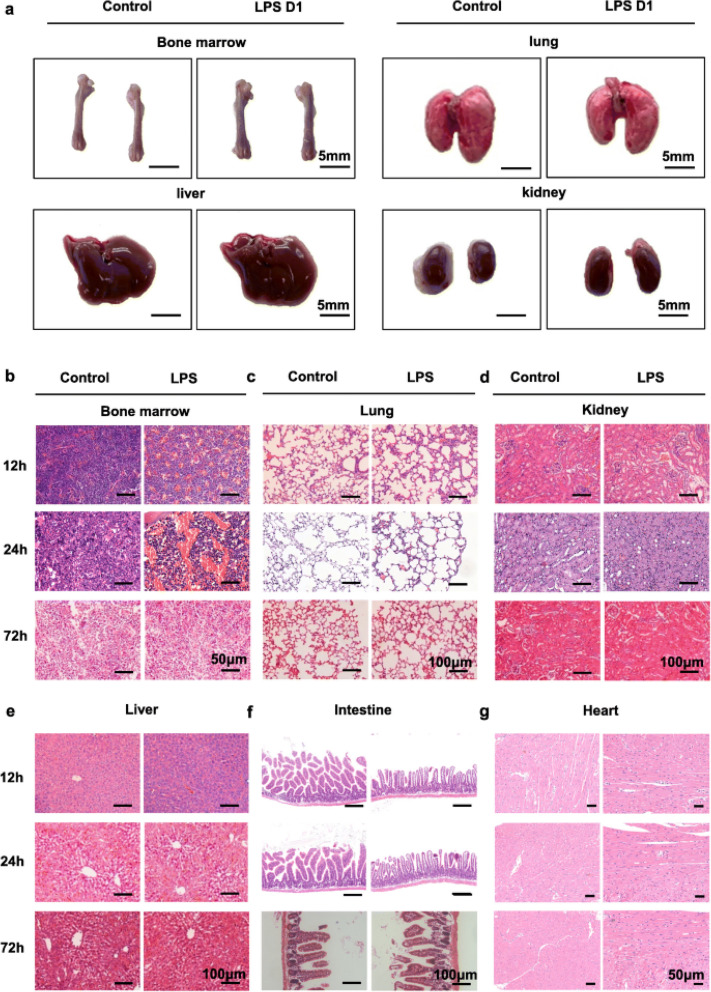
BM undergoes significant histological changes after LPS challenge. **a** Macroscopic images of BM, lung, liver and kidney from 8-week-old control (day 1 after PBS treatment) and LPS D1 (day 1 after LPS treatment) mice. **b, c, d, e, f, g** Histological analysis by H&E staining in the BM (BM) (**b**), lung (**c**), kidney (**d**), liver (**e**), intestine (**f**) and heart (**g**) of mice sepsis model prepared by intraperitoneal injection of LPS at 12 h, 24 h and 72 h after treatment. All representative pictures are verified by independent experiments (n = 3), both control and LPS-treated mice have biological replicates (n > 5)

Correct Fig. 1:

**Fig. 1:**
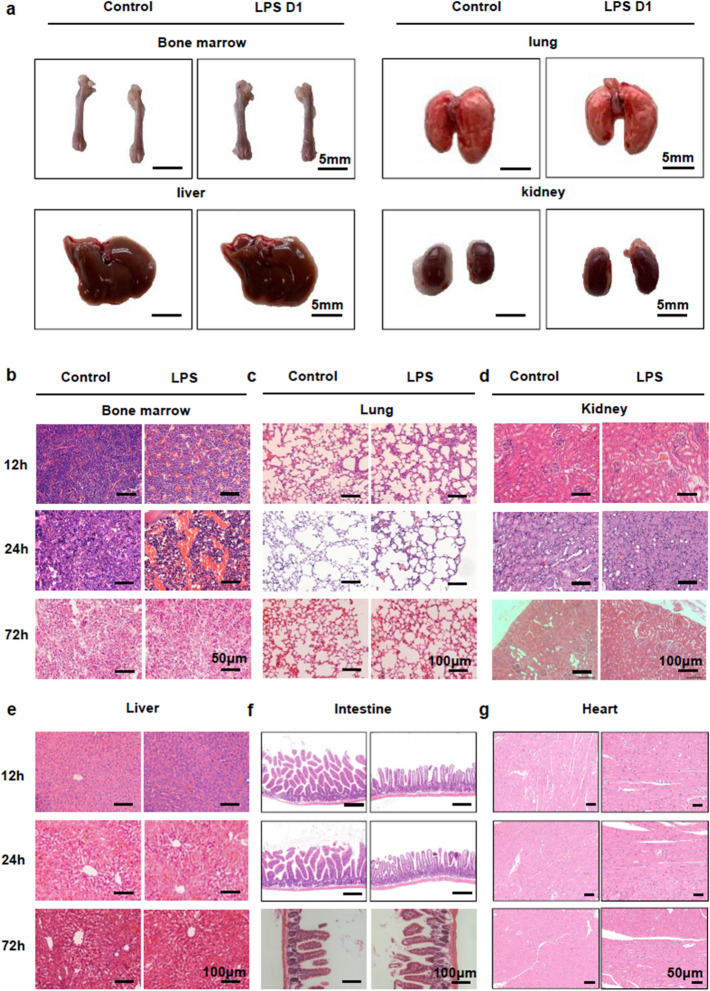
BM undergoes significant histological changes after LPS challenge. **a** Macroscopic images of BM, lung, liver and kidney from 8-week-old control (day 1 after PBS treatment) and LPS D1 (day 1 after LPS treatment) mice. **b, c, d, e, f, g**. Histological analysis by H&E staining in the BM (BM) (**b**), lung (**c**), kidney (**d**), liver (**e**), intestine (**f**) and heart (**g**) of mice sepsis model prepared by intraperitoneal injection of LPS at 12 h, 24 h and 72 h after treatment. All representative pictures are verified by independent experiments (n = 3), both control and LPS-treated mice have biological replicates (n > 5)

Incorrect Fig. 2:

**Fig. 2:**
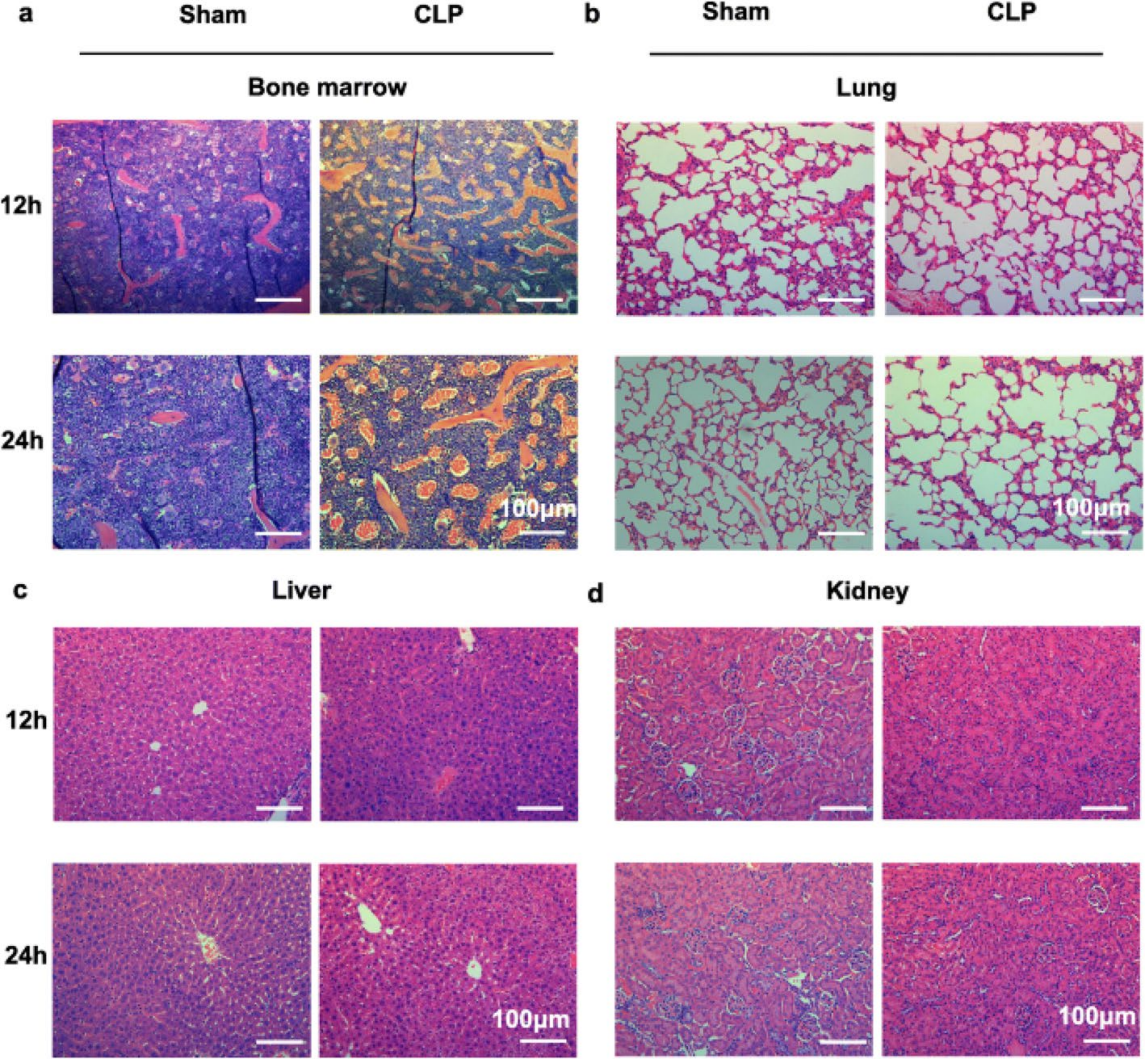
BM in CLP model exhibits similar changes with LPS stimulation. **a, b, c, d** Pictures of H&E staining in the BM (**a**), lung (**b**), liver (**c**) and kidney (**d**) of mice sepsis model prepared by cecal ligation and puncture (CLP) at 12 h and 24 h after operation. All representative pictures are verified by independent experiments (n = 3), both sham-treated and CLP-treated mice have biological replicates (n > 5)

Correct Fig. 2:

**Fig. 2:**
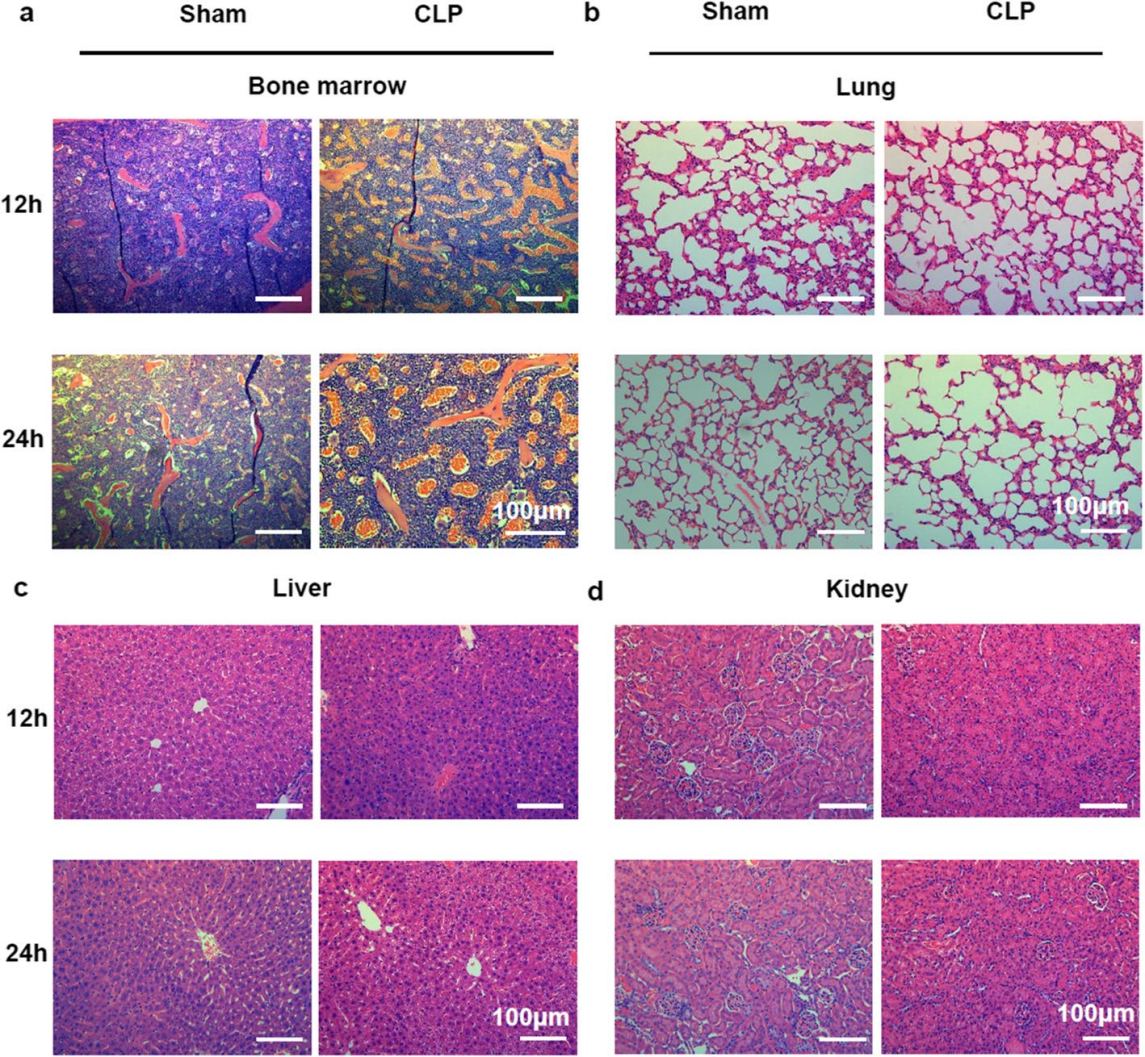
BM in CLP model exhibits similar changes with LPS stimulation. **a, b, c, d** Pictures of H&E staining in the BM (**a**), lung (**b**), liver (**c**) and kidney (**d**) of mice sepsis model prepared by cecal ligation and puncture (CLP) at 12 h and 24 h after operation. All representative pictures are verified by independent experiments (n = 3), both sham-treated and CLP-treated mice have biological replicates (n > 5)

Incorrect Fig. 3:

**Fig. 3:**
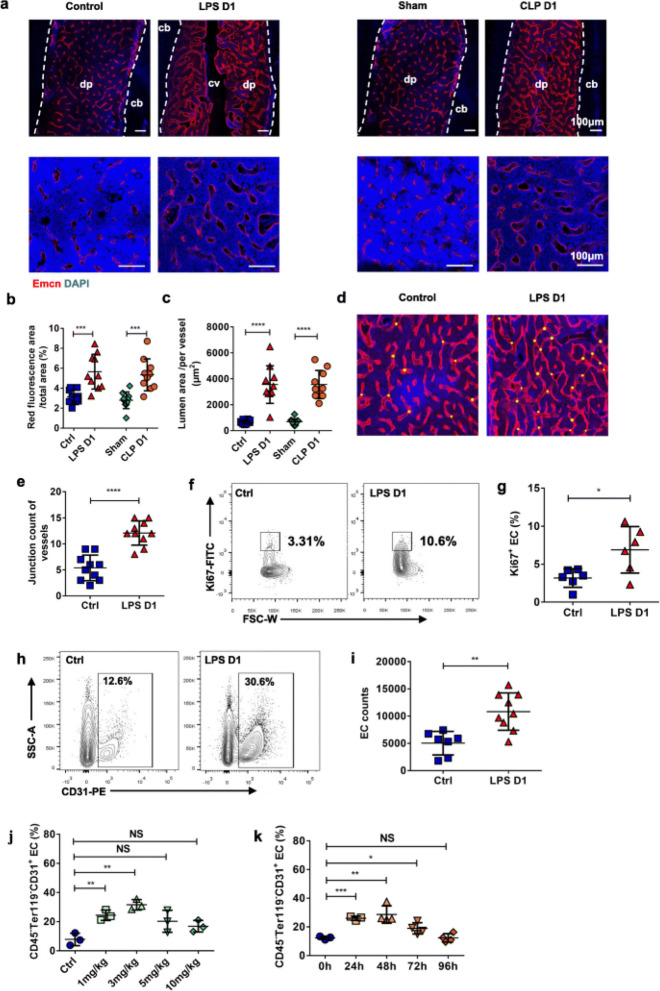
Sepsis alter the BM Vasculature remodeling. **a** Representative images of the femur diaphysis stained with anti-endomucin (Emcn) antibody and 4′,6-diamidino-2-phenylindole (DAPI). Control (day 1 after PBS treatment), LPS D1(day 1 after LPS treatment), Sham (day 1 after sham operation), CLP D1 (day 1 after CLP procedure). Diaphysis (dp), compact bone (cb), central vein (cv). **b, c** Quantification of BM vessels on confocal images of the femur diaphysis showed in (**a**). Ten mice each group, 3 sections per mouse. **d** Visualization of femur vessels junctions (yellow dot) in histological images. **e** Quantitative analysis of junction count of BM vessels showed in (**d**) between control (n = 10) and LPS-treated (n = 10) mice. Three sections per mouse. **f** Ki67 staining for detecting proliferation rates of BM ECs. **g** Percentage of Ki67^+^ BM ECs in controls (n = 6) and treated mice 1 day after LPS injection (n = 6). **h** Representative figures shown the percentage of CD45^−^ Ter119^−^ CD31^+^ ECs in BM. **i** Percentage of CD45^−^ Ter119^−^ CD31^+^ BM ECs in controls (n = 7) and treated mice 1 day after LPS injection (n = 9). **j** Changes of percentage in BM ECs at different concentrations of LPS administration. Three mice in each treatment. Control (Ctrl, PBS treatment). **k** Changes of percentage in BM ECs at different time after 10 mg/kg LPS administration. Three mice in oh and 24 h, 4 mice in 48 h, 72 h, 96 h. All data represent as means ± s.d. *p < 0.05, **p < 0.01, ***p < 0.001, ****p < 0.0001, as determined by Student’s t-test. NS, not significant

Correct Fig. 3:

**Fig. 3:**
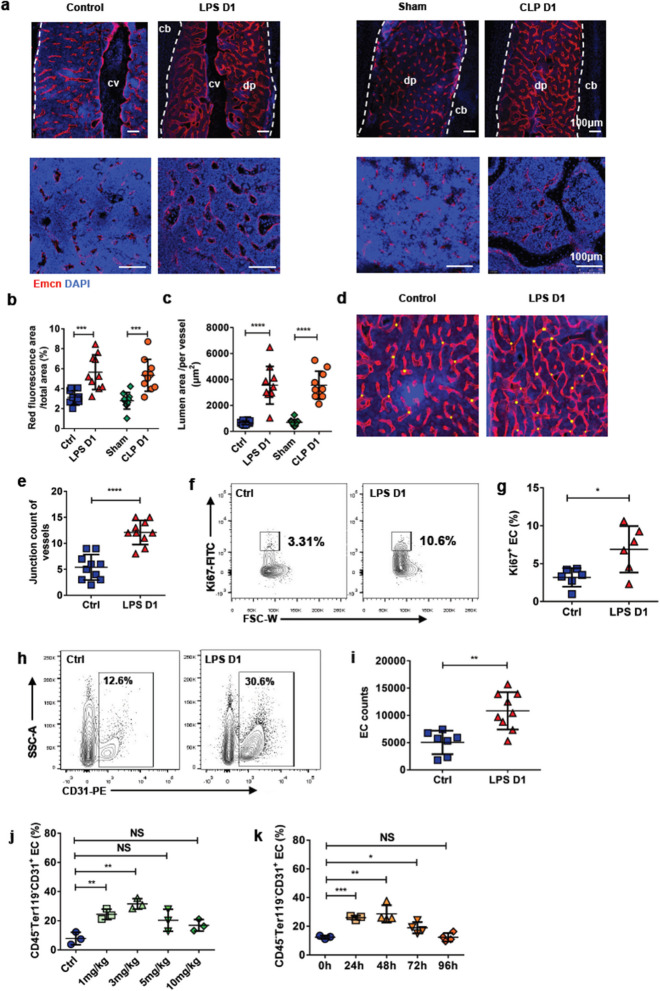
Sepsis alter the BM Vasculature remodeling. **a** Representative images of the femur diaphysis stained with anti-endomucin (Emcn) antibody and 4′,6-diamidino-2-phenylindole (DAPI). Control (day 1 after PBS treatment), LPS D1(day 1 after LPS treatment), Sham (day 1 after sham operation), CLP D1 (day 1 after CLP procedure). Diaphysis (dp), compact bone (cb), central vein (cv). **b, c** Quantification of BM vessels on confocal images of the femur diaphysis showed in (**a**). Ten mice each group, 3 sections per mouse. **d** Visualization of femur vessels junctions (yellow dot) in histological images. **e** Quantitative analysis of junction count of BM vessels showed in (**d**) between control (n = 10) and LPS-treated (n = 10) mice. Three sections per mouse. **f** Ki67 staining for detecting proliferation rates of BM ECs. **g** Percentage of Ki67^+^ BM ECs in controls (n = 6) and treated mice 1 day after LPS injection (n = 6). **h** Representative figures shown the percentage of CD45^−^ Ter119^−^ CD31^+^ ECs in BM. **i** Percentage of CD45^−^ Ter119^−^ CD31^+^ BM ECs in controls (n = 7) and treated mice 1 day after LPS injection (n = 9). **j** Changes of percentage in BM ECs at different concentrations of LPS administration. Three mice in each treatment. Control (Ctrl, PBS treatment). **k** Changes of percentage in BM ECs at different time after 10 mg/kg LPS administration. Three mice in oh and 24 h, 4 mice in 48 h, 72 h, 96 h. All data represent as means ± s.d. *p < 0.05, **p < 0.01, ***p < 0.001, ****p < 0.0001, as determined by Student’s t-test. NS, not significant

Incorrect Fig. 4:

**Fig. 4:**
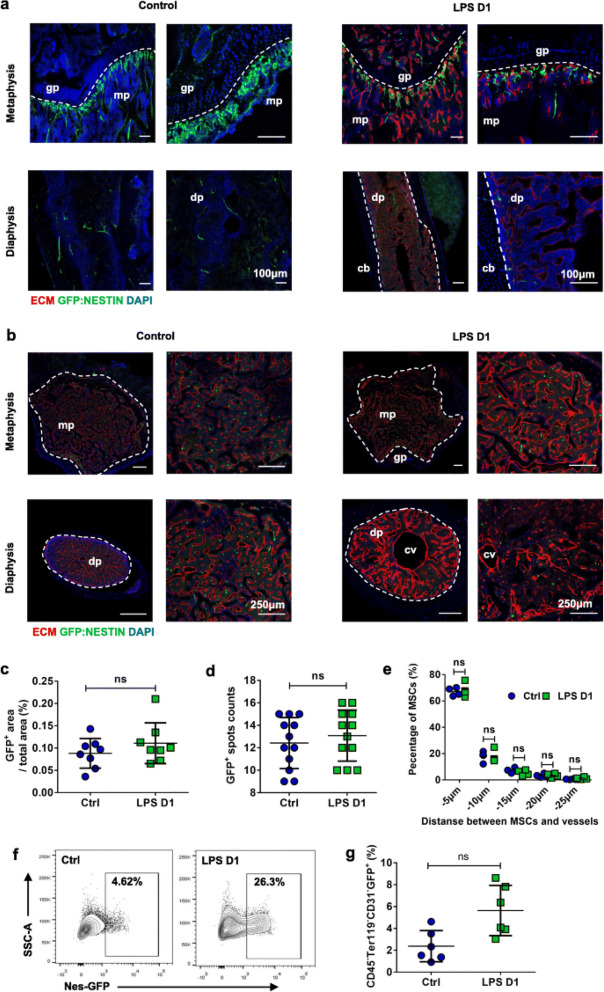
BM MSCs show no significant changes during sepsis. **a, b** Longitudinally (**a**) and transverse-shaved (**b**) confocal images of femur metaphysis and diaphysis stained with anti-Emcn antibody and DAPI in control (PBS treatment) and LPS-treated Nestin-GFP transgenic mice. Diaphysis (dp), metaphysis (mp), central vein (cv), growth plate (gp). **c, d** Quantification of BM GFP + MSCs on enlarged view showed in (**b**). Eight mice each group, 3 sections per mouse. **e** Quantification of distance between MSCs and BM vessels from femur transverse-shaved sections of control (PBS treatment) and LPS-treated Nestin-GFP mice 1 day after intervention. **f** Representative FACS figures showed the percentage of BM MSCs in control (PBS injection) and LPS-treated Nestin-GFP transgenic mice 1 day after intervention.** g** Percentage of CD45 − Ter119 − CD31 − GFP + MSCs in control (n = 6) and LPS treated (n = 6) mice 1 day after intervention. All data represent as means ± s.d. NS, not significant

Correct Fig. 4:

**Fig. 4:**
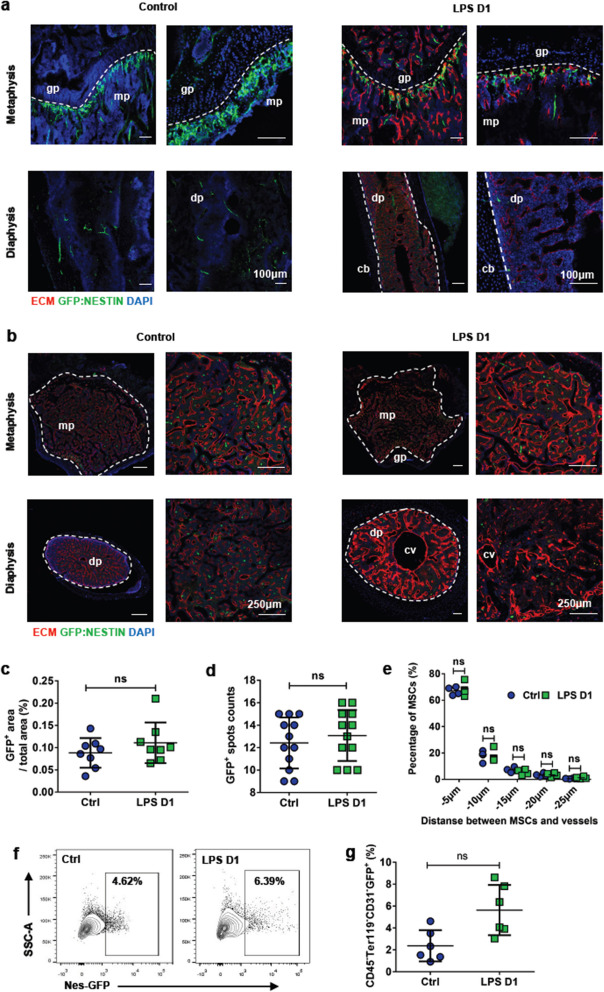
BM MSCs show no significant changes during sepsis. **a, b** Longitudinally (**a**) and transverse-shaved (**b**) confocal images of femur metaphysis and diaphysis stained with anti-Emcn antibody and DAPI in control (PBS treatment) and LPS-treated Nestin-GFP transgenic mice. Diaphysis (dp), metaphysis (mp), central vein (cv), growth plate (gp). **c, d** Quantification of BM GFP + MSCs on enlarged view showed in (**b**). Eight mice each group, 3 sections per mouse. **e** Quantification of distance between MSCs and BM vessels from femur transverse-shaved sections of control (PBS treatment) and LPS-treated Nestin-GFP mice 1 day after intervention. **f** Representative FACS figures showed the percentage of BM MSCs in control (PBS injection) and LPS-treated Nestin-GFP transgenic mice 1 day after intervention.** g** Percentage of CD45 − Ter119 − CD31 − GFP + MSCs in control (n = 6) and LPS treated (n = 6) mice 1 day after intervention. All data represent as means ± s.d. NS, not significant

The original article [1] has been updated.
